# Tackling mosquito-borne viruses in the Region of the Americas

**DOI:** 10.2471/BLT.23.020723

**Published:** 2023-07-01

## Abstract

The spread of mosquito-borne diseases in the Region of the Americas is focusing attention on an innovative approach to vector control. Gary Humphreys reports.

When Luciano Moreira’s daughter came down with a fever in January of 2017, Moreira was more than concerned. “She was prostrate with a fever and had joint pain, and we’d just come out of a year of elevated chikungunya and dengue cases, not to mention Zika,” says the 56-year-old resident of Belo Horizonte, Brazil, a city of 2.7 million people.

Two days after her symptoms started, the 19-year-old was diagnosed with dengue.

As a scientist specializing in mosquito-borne diseases at the Oswaldo Cruz Foundation (Fiocruz) in Rio de Janeiro, Moreira was well acquainted with what the disease – a mosquito-borne viral infection caused by one of four closely related viruses – could do.

“Symptoms range from the barely noticeable to life threatening,” he says. “On an anxiety scale of 1 to 10, I would say my wife and I were at 7.”

For many Brazilians, and nationals of other countries in the Region of the Americas, the needle on the anxiety scale has been moving steadily higher in the past 18 months with the intensification and spread of mosquito-borne viral infections.

“A total of 3.1 million cases (suspected and confirmed) of arboviral (arthropod- associated) diseases were reported in the region in 2022,” says Thais dos Santos, advisor on surveillance and control of arboviral diseases at the Pan American Health Organization (PAHO). “Just over 2.8 million of those were dengue cases, up from 1.2 million in 2021, while 273 685 were chikungunya cases, up from 137 025 cases in 2021.”

According to dos Santos, there are indications that 2023 will see the trends continue. The rise in chikungunya cases is particularly alarming, 2023 seeing 214 719 cases in the first quarter – a 128% increase relative to first quarter 2022 when 94 250 cases were reported. Paraguay has been hardest hit, reporting 138 730 cases by the end of April, compared to just 15 cases in the same period in 2022.

“The severity of the Paraguay outbreak has been compounded by the number of hospitalizations and deaths, including the deaths of four newborns, and a troubling association with acute meningoencephalitis,” says dos Santos. “This is a reminder that chikungunya can also be fatal, as well as causing protracted and often devastating disability.”

While it is widely accepted that the surge in mosquito-borne disease is linked to the increased distribution of *Aedes* mosquitoes (mainly *Aedes aegypti* – the principal vector for both diseases) which are spreading as a result of warmer temperatures and increased humidity, dos Santos cautions against oversimplification.

“Despite systematic implementation of vector control […] transmission continues.”

Raman Velayudhan

“Prior to 2022, reported prevalence for both of these diseases was characterized by significant variation at times, reflecting a couple of human population-level events, namely the COVID-19 pandemic and the Zika outbreaks of 2015 and 2016,” she says, adding that the overall trend was downwards or flat until 2022.

Haroldo Bezerra, a vector-borne disease expert at PAHO, takes a similar view. “These are different viruses that react differently with their vectors, the human populations they infect and with each other, all of which needs to be taken into consideration when viewing epidemiological data.”

Among the differences Bezerra underlines is the fact that chikungunya infection results in lifetime immunity, whereas dengue, with its four serotypes, can be contracted again and again. He also notes that dengue has probably been in the Americas since the 17th century, while chikungunya only arrived in the region at the end of 2013.

The one constant in this complex epidemiological interplay are the mosquitoes that carry the viruses. And with the exception of work on dengue vaccines, it is the mosquitoes that have been the focus of response efforts.

Those efforts have taken the form of vector control, with chemical approaches ranging from indoor residual spraying to outdoor fogging, and non-chemical approaches aimed at eliminating or reducing mosquito breeding sites, for example by reducing sources of standing water.

Ideally, such interventions are combined in what has come to be known as integrated vector management. In reality, that ideal has rarely been achieved, with real-world barriers that include the lack of financial, human and infrastructural resources, and fragmented governance and coordination in many of the countries most affected.

Even single components of vector control are hard to implement, a point made by Dr Diana Rojas Alvarez, co-lead of the Global Arbovirus Initiative and technical lead for Zika and chikungunya in the World Health Organization’s (WHO) Emergencies Programme, who grew up in Colombia.

“In my community, piped water was unreliable,” she says. “So people tended to store water in containers when the faucet was flowing. Those containers then sat uncovered around the house and mosquitoes laid eggs in them.”

Even in settings where vector control interventions are rigorously implemented, challenges arise. “In Singapore, we have seen that despite systematic implementation of vector control which includes fines on households for non-adherence, dengue transmission continues, probably as a result of cryptic breeding in undiscovered sites,” says Raman Velayudhan, who coordinates WHO’s dengue and arbovirus work in the Global Neglected Tropical Diseases Programme.

Partly with a view to meeting these challenges, in 2016, Singapore’s National Environment Agency started testing a different approach which involved releasing male mosquitos infected with the *Wolbachia* bacterium into targeted areas as part of mosquito population suppression efforts. According to agency reports, the initiative has resulted in a dramatic fall in the *Aedes aegypti* population and human cases of dengue at study sites in Yishun and Tampines, Choa Chu Kang and Bukit Batok towns.

Interest in *Wolbachia* as a vector control tool is not new, dating back to breakthroughs made in 2009 by Scott O’Neill and a team of scientists at Monash University in Melbourne, Australia. The team discovered that *Wolbachia* not only prevents the replication and transmission of certain viruses (including dengue and chikungunya), but also prevents male mosquitoes from reproducing.

“These discoveries became the basis of two distinct vector control approaches,” explains O’Neill, who is now chief executive officer of the World Mosquito Program (WMP), a not-for-profit, nongovernmental organization supporting *Wolbachia*-based vector control projects worldwide.

The first approach is mosquito population suppression (as used in Singapore), whereby *Wolbachia*-infected males are released, mate with uninfected females who then produce eggs that will not hatch. The second is mosquito population replacement, whereby *Wolbachia*-infected females are released to breed, passing the bacteria into the next generation.

“Over the next 10 years we will be able to protect around 70 million people.”

Luciano Moreira

The WMP has focused its efforts on the latter. “There are a number of advantages with replacement, one of which is sustainability,” O’Neill explains. “When you reach 50% *Wolbachia* coverage of the mosquito population, the propagation process becomes self-sustaining. That’s a big advantage of the intervention – you only have to do it once.”

According to O’Neill, 13 countries have so far implemented replacement programmes, many at scale, and in almost all settings the results have been impressive. In Yogyakarta, for example, a city of half a million people on the island of Java, Indonesia, *Wolbachia*-infected mosquitoes were released between March and December of 2017, resulting in a 77% reduction in dengue incidence, and an 86% reduction in dengue hospitalizations three years after the initiative, as reported in the June 2021 issue of the *New England Journal of Medicine*.

In the face of growing evidence of efficacy, and community demand stimulated by heightened perception of infection risk – notably in urban areas – many policy makers are now significantly ramping up their *Wolbachia* programmes.

Brazil is a case in point. With funding from the health ministry, Fiocruz is already implementing replacement initiatives in the cities of Rio de Janeiro, Niterói, Campo Grande, Belo Horizonte, and Petrolina covering a total of 3.2 million people in target areas.

The projects rely on the release of adult mosquitoes from tubes carried in vans that follow predetermined routes and/or the placement of *Wolbachia*-infected eggs in small, nutrient-filled phials in target areas.

“The emphasis has been on simplicity and sustainability,” explains Luciano Moreira who, along with his Fiocruz responsibilities, is lead researcher for the WMP in Brazil. The releases take four to six months, and one month after initiation, traps are installed to monitor the prevalence of *Wolbachia* in the mosquito population.

Moreira reports being inundated with requests from some 40 cities across country. “Until recently, we have not been able to respond because of our inability to provide infected eggs,” he says.

That may be about to change. In April, Fiocruz and the WMP announced a partnership aimed at massively expanding the supply of *Wolbachia*-infected mosquitoes for distribution in selected urban centres. The partnership will support the building of a facility that will have the capacity to produce up to 100 million *Wolbachia*-infected mosquito eggs per week.

“One hundred million eggs per week means about 5 billion eggs per year,” says Moreira. “Based on health ministry estimates of populations at risk in high priority areas, and current resources available for distribution, we estimate that over the next 10 years we will be able to protect around 70 million people.”

For Scott O’Neill, whose first field work on *Wolbachia* as a vector control tool dates back to 2011, the work going on in Brazil marks a significant milestone. While stressing the need to embed the intervention within comprehensive approaches to vector control, he is excited to see what comes next. “I think we might have reached take-off,” he says.

**Figure:**
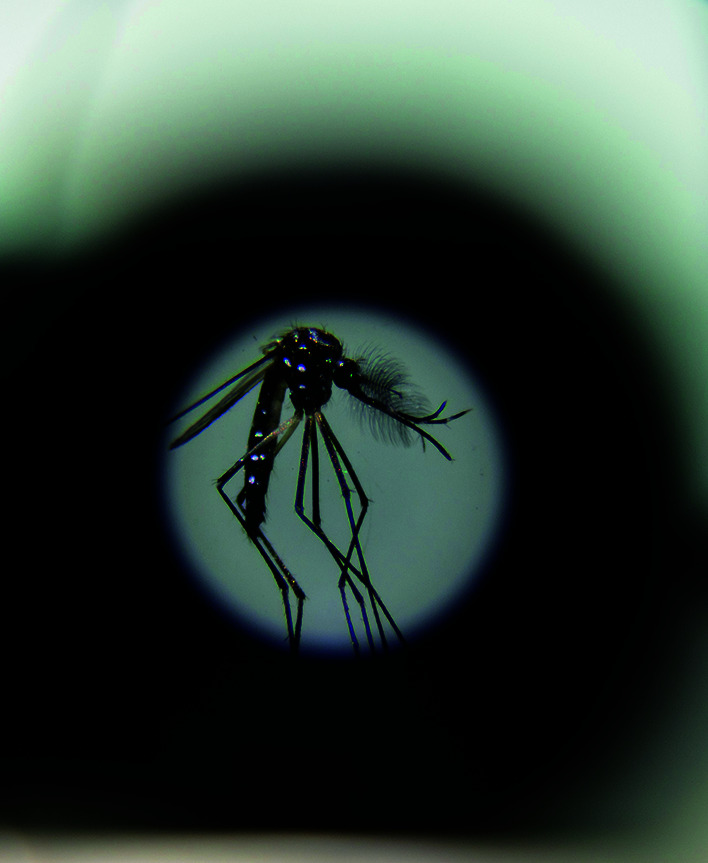
Examining an ***Aedes aegypti*** mosquito in a Fiocruz lab

Courtesy of the Oswaldo Cruz Foundation

**Figure:**
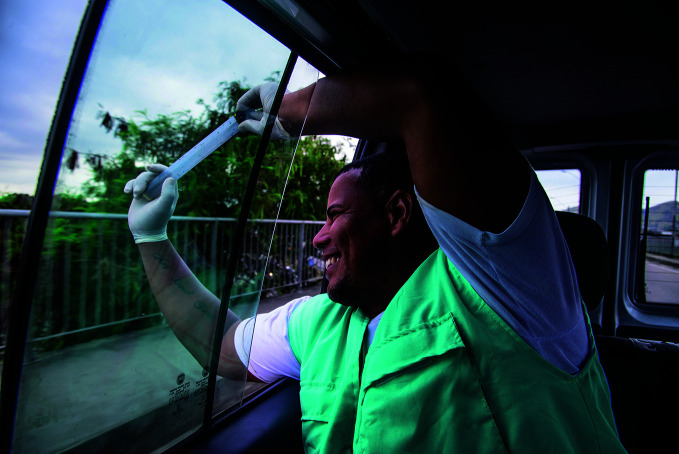
Release of ***Wolbachia***-infected ***Aedes aegypti*** in Rio de Janeiro

Courtesy of the Oswaldo Cruz Foundation

